# Effects of supplemental light on tomato growth and the mechanism of the photosystem II apparatus

**DOI:** 10.1371/journal.pone.0267989

**Published:** 2022-05-05

**Authors:** Xiaoling Yang, Haibo Sun, Mingyan Hua, Lanfang Song, Zhongpin Du, Yana Tong, Hongying Ma, Zhiwen Song

**Affiliations:** 1 Tianjin Academy of Agricultural Sciences, Tianjin, China; 2 Qinghai Academy of Agriculture and Forestry Sciences, Xining, Qinghai Province, China; University of Hyderabad School of Life Sciences, INDIA

## Abstract

The addition of supplemental light (SL) is an effective way to offset insufficient lighting. Although it is commonly believed that SL increases leaf photosynthesis and therefore improves yield and fruit flavor, the mechanism underlying the effects of SL on the photosystem II (PSII) apparatus remains unclear, and SL leads to high energy consumption. In order to save energy, we investigated the physiological status of the PSII apparatus, plant growth parameters and fruit parameters under two types of overhead SL with a low daily energy consumption of 0.0918 kWh m^-2^. The results showed that SL significantly increased the leaf chlorophyll content from full unfolding to yellowing. However, a remarkable increase in the absorption flux per cross-section (ABS/CS), the quantum yield of electron transport (φ_Eo_) and the performance index (PI_abs_) was observed only in a relatively short period of the leaf life cycle. SL also enhanced the fruit yield and quality. The obviously increased ΔV_K_ and ΔV_J_ components of the chlorophyll fluorescence induction kinetic (OJIP) curve, along with the significantly decreased PI_abs_ from days 40–60 after unfolding in the SL-treated groups, resulted in more rapid leaf aging and earlier fruit ripening compared with the control plants (CK). Therefore, an energy-friendly SL strategy can alter the physiological status of the PSII apparatus, affecting yield and fruit quality and maturity.

## Introduction

Tomato (*Solanum lycopersicum*) is one of the most popular vegetable species in the world, and its year-round production is made possible through greenhouses. To obtain good-tasting fruits and high yields, a daily light integral (DLI) of 20–30 mol m^-2^ day is suggested [1, 2]. In regions that receive low amounts of solar radiation and have short days, such as the northern USA, China and northern Europe, off-season greenhouse-grown tomato fruits have a poor reputation in terms of taste and flavor compared to those of in-season field-grown tomato fruits [3].

Supplemental light (SL) in greenhouse production can greatly offset insufficient amounts of light [4, 5]. Heuvelink et al. [6] and Gómez et al. [7] reported that applying heating and SL to the production of year-round greenhouse-grown tomato improved yields and fruit quality and that these techniques are widely adopted in northern climates. More than 2000 ha of greenhouse space in the Netherlands are equipped with overhead high-pressure sodium (HPS) lamps, and 15% of tomato growers and 10% of cucumber growers in Canada use SL during winter [8]. The most common light source used in SL is an overhead HPS lamp with emission wavelengths ranging from 400 nm to 700 nm, with a peak intensity at 500–650 nm [9, 10]. Constituting another major SL light source, light-emitting diodes (LEDs) are preferable because they can be designed to emit narrow-spectrum light for specific crops [11, 12] and therefore have increased energy efficiency [13]. Kuijpers et al. [14] reported that SL with LED resulted in a 30% carbon emission reduction and a 9% yield increase in comparison with that resulting from the use of HPS lamps.

Previous studies have shown that SL enhances the light absorption of plants as well as CO_2_ absorption and assimilation via increased photosynthesis, which ultimately increases yield. SL can also effectively regulate crop growth and accelerate maturity [15, 16]. Joshi et al. [17] observed a 3.5–5.7-fold increase in the leaf photosynthetic rate of inner canopy foliage and a 30% yield increase when intracanopy LED lighting was applied to pepper. Li et al. [18] applied supplemental intracanopy LED illumination to tomato plants and also observed a significant enhancement in both the stomatal conductance and the photosynthetic capacity for carbon absorption/assimilation in the leaves in the lower and middle canopies; the final yield increased by 8.7%. Lu et al. [19] observed that photosynthesis of tomato could be stimulated by SL, and there was a positive linear relationship between the fruit fresh weight and days of SL application. However, it is still not clear whether SL improves photosynthesis throughout the entire life cycle of plant leaves or merely at a certain stage. It is also unclear whether SL affects the life cycle of leaves.

Even with significant benefits, SL is known to consume a relatively large amount of energy. Tewolde et al. [20] applied a photosynthetic photon flux density (PPFD) of 165 μmol m^-2^ s^-1^ of red–blue (RB)-LED light (with a daily 10-h photoperiod) to tomato, which increased the yield by 24%, but the electric energy consumption reached 1.2 kWh m^-2^ day^-1^. Lu and Mitchell [8] suggested that it is more reasonable to design an SL system with a specific PPFD and duration based on the DLI of solar radiation and crop needs. In West Lafayette (United States), an SL of more than 10 mol m^-2^ day^-1^ of DLI was reported to meet the 25 mol m^-2^ day^-1^ DLI requirement of tomato growth between September and December. However, even with a 33% increase in yield, such an SL strategy consumes more than 3 kWh m^-2^ day^-1^ [4]. Katzin et al. [9] reported that SL installation in greenhouses caused an increase of 2 and 10 times in electricity consumption in the Netherlands and Canada, respectively. Gómez and Mitchell [7] reported that 10–30% of winter electricity consumption in the northern United States was attribute to greenhouses, among which 60% of the power was used for SL. In short, SL increases yield but is also highly energy consuming.

The existing strategy in China for overwintering under SL involves the application of a relatively low amount of radiation, with a short daily SL duration of approximately 3–5 h, resulting in low SL energy consumption. To determine the benefit of an energy-friendly SL strategy, this paper investigated an SL strategy involving less than 0.1 kWh m^-2^ day^-1^ of energy consumption. We investigated these effects on the physiological status of the photosynthetic apparatus in tomato plants and elucidated its effect mechanism on yield and fruit quality and maturity.

## Materials and methods

### Plant material and growth conditions

Seeds of Kaide 6810 tomato (Xinfu Agricultural Company, Beijing, China) were sown in 50-cell plug trays with substrate consisting of vermiculite, turf soil and perlite (1/1/1) in a greenhouse of the Modern Agriculture Innovation Center (Wuqing, Tianjin, China) on September 16, 2019. The resulting seedlings were transplanted into soil whose available N content was 311.65 mg kg^-1^, P_2_O_5_ content was 536.05 mg kg^-1^, K_2_O content was 1161.3 mg kg^-1^ and organic matter content was 59.49 g kg^-1^ on October 16. The planting row spacing was 90 cm×45 cm, and the density was 2.5 plants m^-2^. A total of 5 trusses were left on each plant, and 3, 4, 4, 5, and 5 fruits from the first to 5^th^ trusses, respectively, were kept on the plants, for which harvest began on February 26, 2020 and ended on April 9, 2020. Commercial fertilizers (16-8-34 or 20-20-20 NPK) were applied at 48 kg ha^-1^ each time, and a total of 241 kg ha^-1^ was applied in rotation throughout the growing season. The highest temperature in the greenhouse was approximately 28–32°C during the daytime, and the lowest temperature was 8–9°C during the night on sunny days.

### Lighting treatment

The test plot was divided into nine 3.6×6.8 m blocks separated by a hanging perpendicular movable 2.5 m×7 m black nonwoven fabric curtain between blocks to prevent light pollution. Each of the following 3 different treatments was applied to 9 blocks, and each treatment was repeated 3 times in accordance with a randomized block design.

- T1: Plants exposed to sunlight and SL provided by six overhead 120±5 W HPS lamps in each block.
- T2: Plants exposed to sunlight and SL provided by six overhead 120±5 W RGB-LED lamps in each block.
- CK: Plants exposed only to sunlight, without SL.

**Fig 1:**
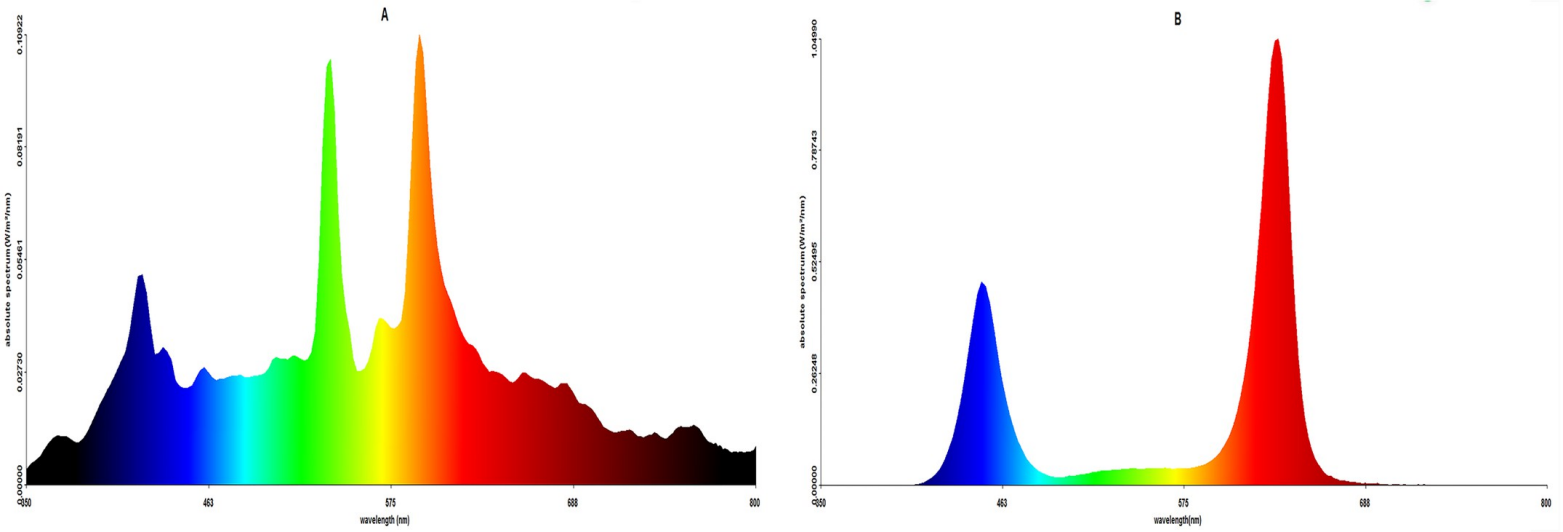
Spectra of two types of lamps. (A, HPS; B, LED).

**Fig 2:**
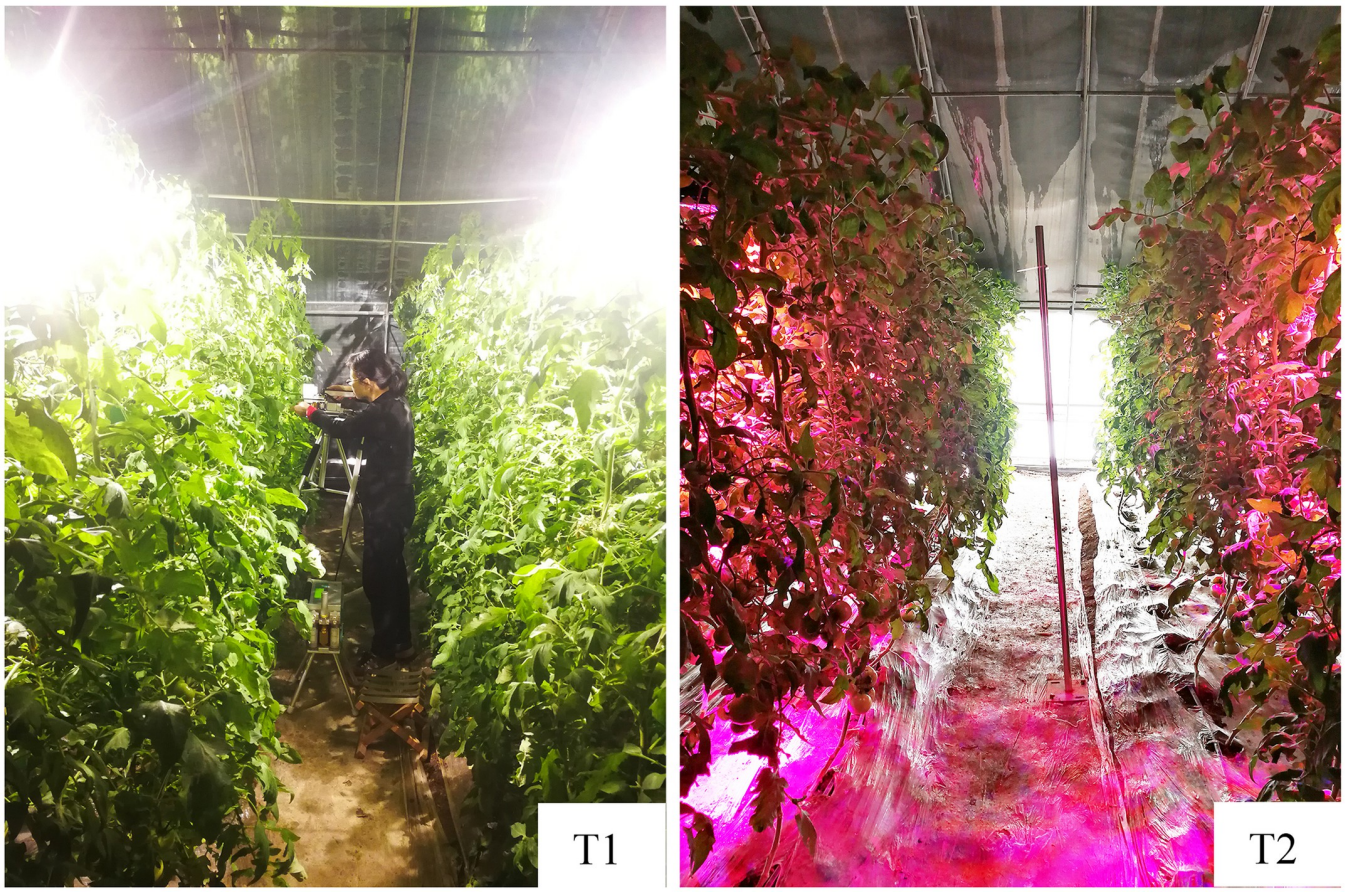
Treatments with supplemental light. (T1, HPS; T2, LED).

The HPS lamps were obtained from Rongtai Guangyuan Co., Ltd., Henan, China, and the LED lamps with customized emission spectra were obtained from Qilian Technology Co., Ltd., Tianjin, China. The spectra of two types of lamps measured by PLA-20 (Everfine Photo-E-Info Co., Ltd., China) are shown and described in Figs 1 and 2 and Table 1, respectively. From October 30, 2019, to February 4, 2020, SL was applied daily from 16:30 to 19:30. In general, the solar radiation duration was approximately 7–8 h in the CK every day, while the photoperiod was approximately 10–11 h in the SL-treated groups.

**Table 1:** Light quality of the two types of lamps used.

Type	Light component	R/G/B ratio	PPFD μmol m^-2^ s^-1^ (30 cm below the lamp)
HPS	16% red+80% green+4% blue	4/20/1	143
LED	50%r ed+40% green+10% blue	5/4/1	176

### Measurements

#### Chlorophyll fluorescence and chlorophyll fluorescence induction kinetic (OJIP) curve parameter measurements

Chlorophyll fluorescence was analyzed with Handy PEA (Hansatech Instruments, Ltd., Norfolk, UK). For each experimental treatment, the first leaves above the first truss fruit from 10 random plants were measured at the same position and then averaged. The first measurement was performed after most leaves had just completely unfolded and was recorded as the measurement for the 1^st^ day, and then the measurements were repeated on the 10^th^ day, 20^th^ day, 40^th^ day, and 60^th^ day. Before the measurements, the leaflets were clamped into the leaf chamber of the instrument to adapt to the dark for 30 minutes. Then, 3500 μmol m^-2^ s^-1^ PPFD of saturated light was applied to determine the OJIP.

The intensity of chlorophyll fluorescence was recorded in arbitrary units. Those were transformed into units of the relative variable chlorophyll fluorescence (V_t_) via double normalization to that of the initial level, minimum level (F_0_) and to the maximum (F_m_) level. Taking the V_t_ of CK at day 1 as the reference (V_t_ref_), the ΔV_t_ of the different treatments was calculated via V_t_ − V_t_ref_ at the corresponding moment of the induction time, and different ΔV_t_ curves were generated.

The characteristic points of the OJIP curves were used to calculate specific characteristics of the light reactions of photosynthesis according to the OJIP test algorithms described by Strasser et al. [21, 22]. The analyzed parameters are described in Table 2.

**Table 2:** Definitions of measured and calculated chlorophyll a fluorescence parameters.

Fluorescence parameters	Description
F_0_	Minimum fluorescence, when all PSII RCs are open
F_m_	Maximum recorded fluorescence at the P step when all RCs are closed
F_J_	Fluorescence at the J step (2 ms) of the O-IP curve
F_I_	Fluorescence at the I step (30 ms) of the OJIP curve
ABS/CS	Absorption flux (of antenna chlorophyll) per CS
ABS/RC = ABS/RC = M_0_ × (1/V_J_) × (1/φ P_0_)	Absorption flux (of antenna chlorophyll) per RC
RC/CS	
RE_0_/RC = M_0_ × (1/V_J_) × (1 − V_I_)	Flux of electrons reducing the terminal electron acceptor at the PSI acceptor side, per RC
φ_Po_ = TR_0_ /ABS = [1 − (F_0_ /F_m_)]	Maximum quantum yield of primary photochemistry
φ_Eo_ = ET_0_ /ABS = φ_Po_ × ψ_E0_	Quantum yield of electron transport (at *t* = 0)
δ_R0_ = RE_0_ /ET_0_ = (1 − V_I_) / (1 − V_J_)	Efficiency/probability with which an electron from intersystem electron carriers moves to reduce terminal electron acceptors at the PSI acceptor side (RE)
PI_abs_ = γ_RC_ / (1 − γ_RC_) × φ_Po_ /(1 −φ_Po_) × ψ_Eo_ /(1 − ψ_Eo_)	Performance index of PSII based on absorption

#### Plant growth, yield and chlorophyll content measurements

On January 17, 2020, 20 plants were selected randomly in each treatment group (Fig 2), and then, the height of plants from the base of the stem to the first leaf above the 4^th^ truss fruit and the stem diameter under the third truss fruit were measured. The node of the first flower was counted to determine the number of leaves between the first leaf at the bottom and the first flower node, and the average internode length was the ratio of plant height to the number of leaves. The fresh fruit weight was recorded and aggregated in each treatment.

There is a linear correlation between the chlorophyll content of leaves and the SPAD value [23]. Therefore, the chlorophyll content was measured with a SPAD-502 plus chlorophyll meter (Spectrum Technologies, Inc., USA) 7 times *in situ*, and each measurement was performed on 30 leaves of plants in each block selected randomly every 10 days; only leaves that were completely unfolded to turning yellow were used. The measurements were carried out on the middle part of the terminal leaflet of the first leaf above the first truss fruit.

#### Fruit quality and maturity measurements

The soluble sugar content and the titratable acidity were used as indicators of fruit quality. The soluble sugar content was quantified by anthrone-sulfuric acid assays [24], and the titratable acidity was quantified by the acid-base titration method. Samples were collected randomly from 6 fruits in each block.

The tomato ripening stage is distinguished by fruit color. Thus, fruit color was measured 100 days and 110 days after transplanting, when some fruits just began to change color. The first fruits on the first truss from 15 plants were selected randomly in each block, and the color was determined *in situ* with an NR145 Precision Colorimeter (Sanenshi Technology Co., Ltd., Shenzhen, China). A positive/negative a* value indicates a reddish/greenish color, while a positive/negative b* value indicates a bluish/yellowish color. Each fruit was measured 4 times in different directions, the a* and b* data were averaged, and the ratio of a*/b* was calculated. The mean a*/b* in each block was used as the indicator of fruit color. At the same time, a field survey was conducted to determine the percentage of fruits at different stages of color change.


Percentageofredfruits=numberofplantswithredandripefruits/totalnumberofplants×100%

Percentageofcoloringfruit=numberofplantswithvisiblybutnotcompletelyreddishfruits/totalnumberofplants×100%

Percentageofgreenfruits=thenumberofplantswithcompletelygreenfruits/totalnumberofplants×100%

### Data treatment

Microsoft Excel (Microsoft Corp., Redmond, WA) and SPSS 19.0 were used for statistical analysis and to generate figures. Statistical analysis was performed using one-way ANOVA (for *p < 0*.*05*). Based on the ANOVA results, the least significant difference (LSD) test for main comparisons at the 95% confidential level was applied.

## Results

### Impact of SL on rapid chlorophyll II fluorescence induction kinetic curves and chlorophyll fluorescence parameters

**Fig 3:**
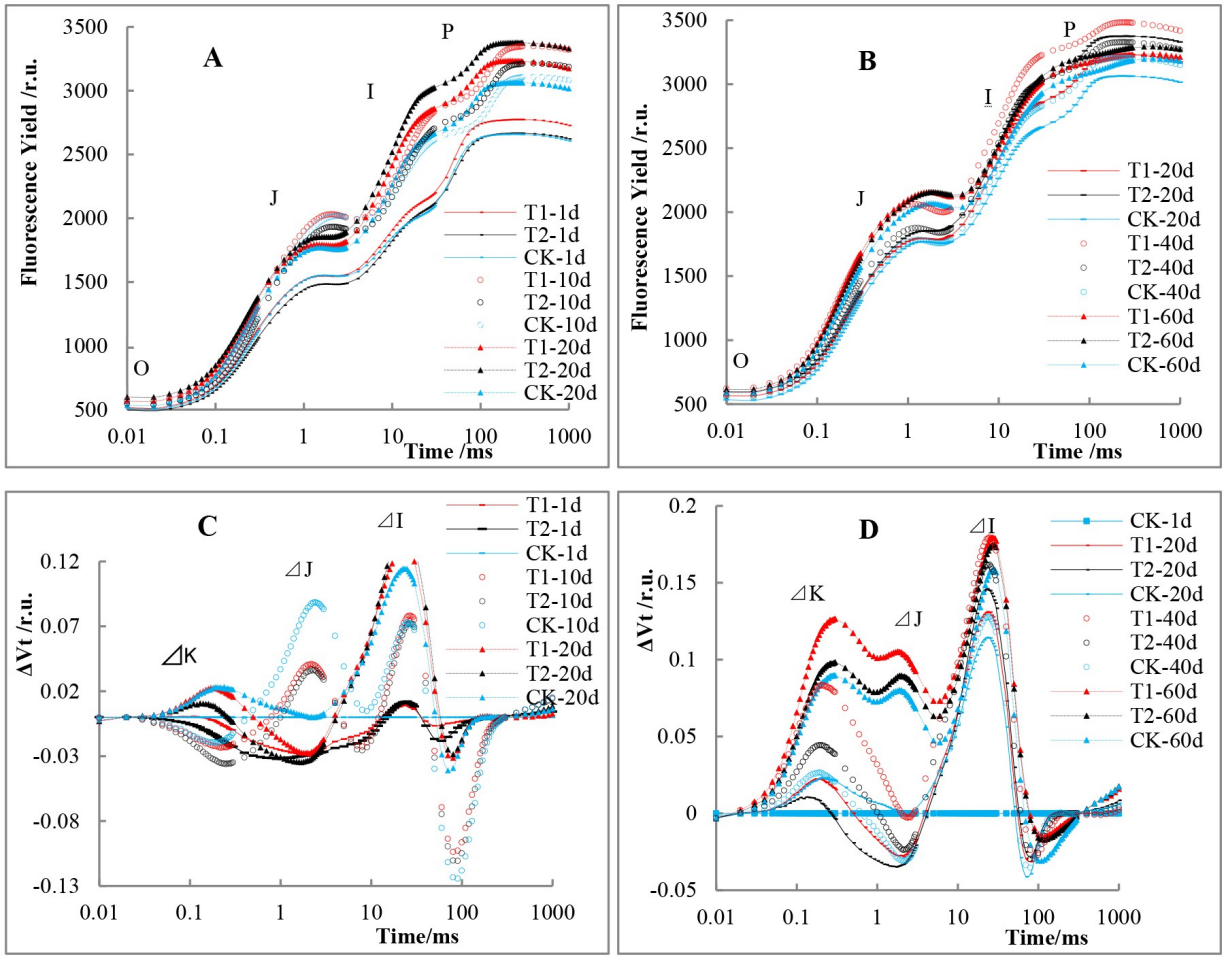
Effects of supplemental light on tomato rapid chlorophyll fluorescence induction kinetic curves. A and B: OJIP curves. C and D: Curves of differential values (ΔV_t_) resulting from subtracting the control value of the first measurement from the treatment values V_t_ [ΔV_t_ = (F_t_ − F_0_)/F_v_ − V_t CK)_]. A and C: Measurements from the first day to the 20^th^ day after the leaves fully unfolded. B and D; Measurements from the 20^th^ day to the 60^th^ day after the leaves fully unfolded. The values are the means (n = 10) ±SEs.

As shown in Fig 3A and 3B, the difference in F_0_ between the SL groups and the CK was not obvious, while the F_m_ values clearly differed between leaves that had completely unfolded and those that were turning yellow. Among them, T1 achieved its maximum F_m_ value on the 10^th^ day after the leaves fully unfolded, T2 achieved its maximum F_m_ value on the 20^th^ day, and the CK achieved this on the 40^th^ day. Regarding the F_m_ values measured at the same points, T1 and T2 showed higher maximum fluorescence yields than the CK did.

The ΔV_t_ (V_t_ − V_t_ref_) curves of the different treatments are shown in Fig 3C and 3D. The peaks at points K, J, and I corresponded to induction times of approximately 0.3 ms, 2 ms, and 30 ms, respectively. The obvious increase in ΔK indicates damage to the activity of the oxygen-evolving complex (OEC), while a clear appearance in ΔJ is related to the blockage of electron transfer from Q_A_ to Q_B_ [25, 26]. On the first day and the 10^th^ day after the leaves fully unfolded, the ΔV_K_ of the SL groups was lower than the ΔV_K_ref_ but it higher than the ΔV_K_ref_ from the 20^th^ day onward (Fig 3C). Compared with the CK, T1 and T2 showed significantly higher ΔV_K_ and ΔV_J_ values from the 40^th^ day to the 60^th^ day (Fig 3D). These results indicate that the OEC activity of the SL groups was lower than that of the CK group, and the inhibition of electron transfer from Q_A_ to Q_B_ was stronger from the 40^th^ day onward.

**Fig 4:**
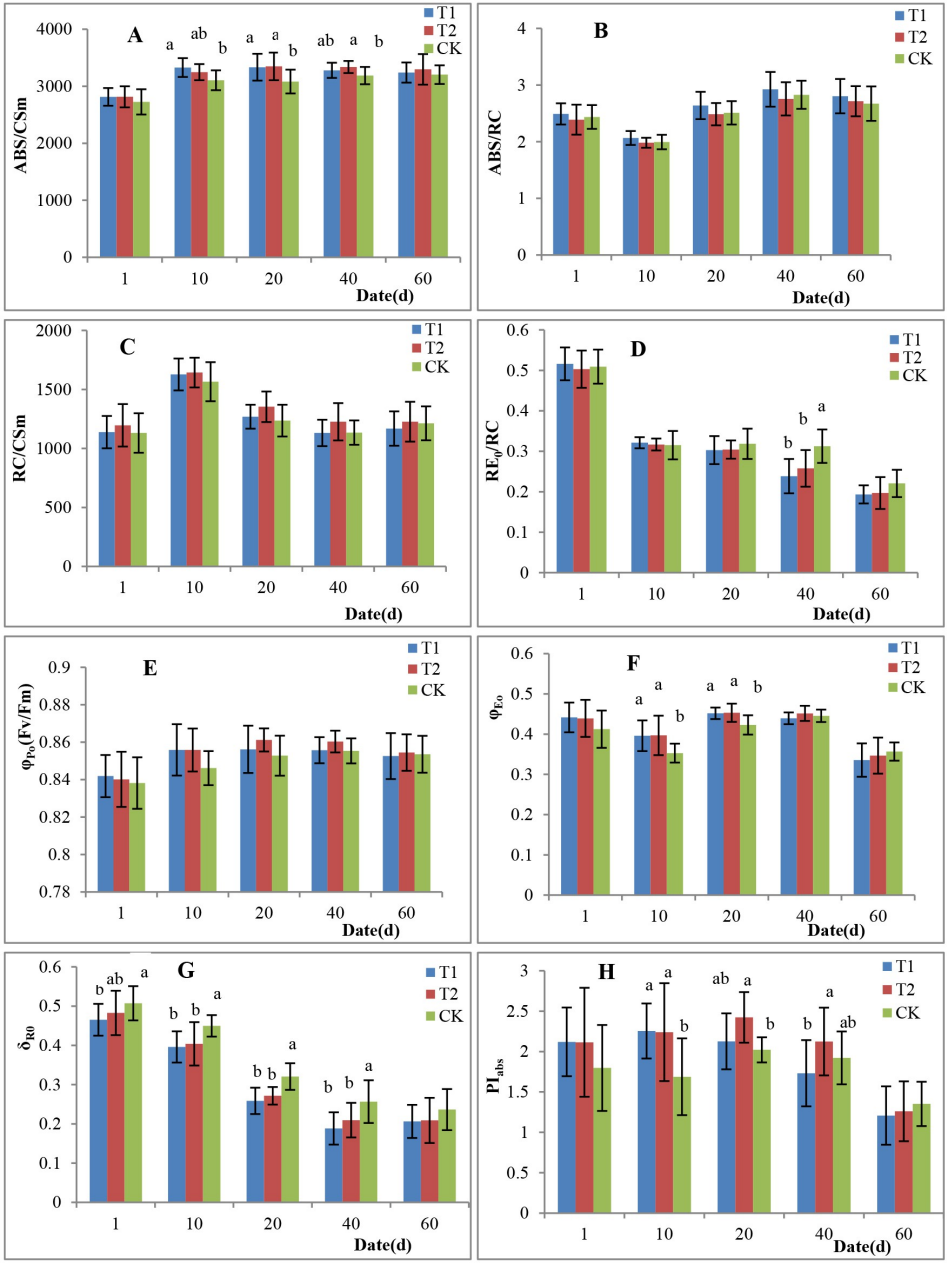
Effects of supplemental light on the chlorophyll fluorescence parameters of tomato leaves. A, ABS/CS (absorption flux per CS). B, ABS/RC (absorption flux per RC). C, RC/CS (density of active PSII RCs per CS). D, RE_0_/RC (electron flux reducing terminal electron acceptors at the PSI acceptor side, per RC). E, φ_Po_ (maximum quantum yield for PSII primary photochemistry). F, φ_Eo_ (quantum yield of electron transport). G, δ_R0_ (efficiency/probability with which an electron from the intersystem electron carriers moves to reduce terminal electron acceptors at the PSI acceptor side). H, PI_abs_ (performance index). The bars (means ±SEs, n = 10) followed by the same letters are not significantly different at *p<0*.*05*.

The absorption flux (of antenna chlorophyll) (ABS) per cross-section (CS) (ABS/CS), ABS/reaction center (RC), RC/CS, and flux of electrons reducing the terminal electron acceptor at the PSI acceptor side (RE_0_)/RC are activity parameters used to assess the photosystem II (PSII) apparatus. The SL treatment increased ABS/CS (Fig 4A). By comparing the SL-treated groups and the CK, we found that the ABS/CS in T1 was significantly higher than that in the CK on the 10^th^ day and the 20^th^ day after the leaves fully unfolded; however, in T2, this occurred on the 20^th^ day and 40^th^ day. There was no significant difference in ABS/CS between T1 and T2, which was approximately 4.5% and 4.8% higher than that in the CK for a total of 6 measurements. The value of ABS/CS was determined by the ABS/RC and RC/CS, both of which were not significantly different among the three treatments (Fig 4B and 4C). However, in terms of values, compared with the other groups, the HPS lamp-treated groups had slightly higher ABS/RC values, and the LED-treated groups had slightly higher RC/CS values. Therefore, we assume that different SL light sources increase light absorption via different mechanisms, e.g., more active RCs in antenna chlorophyll under HPS lamp light and more RCs per CS under LED light. The RE0/RC values were similar among the three treatments, except for the 4^th^ measurement, for which the value was obviously higher in the CK than in T1 and T2 (Fig 4D).

There was no significant difference in the maximum quantum yield of the primary photochemistry (φ_Po_) of PSII or the maximum PSII efficiency (Fv/Fm) among the three treatments (Fig 4E), but the quantum yield of electron transport (φ_Eo_) was different among the treatments (Fig 4F). The φ_Eo_ in T1 and T2 was 7% and 6.5% higher than that in the CK, respectively, with no significant differences in the first measurement; however, significant differences occurred on the 10^th^ day and the 20^th^ day after the leaves fully unfolded, with 12.2% and 6.9% higher φ_Eo_ values in T1 and 12.5% and 7.1% higher φ_Eo_ values in T2, respectively. However, the φ_Eo_ in T1 was the lowest, which was 2.7% and 3.1% lower than those in T2 and 1.4% and 5.9% lower than those in the CK on the 40^th^ day and the 60^th^ day of measurements, respectively. In summary, from when the leaves had completely unfolded to when they turned yellow, the φ_Eo_ values in T1 and T2 were 3.7% and 4.9% higher than that in the CK, respectively. δ_R0_ is the efficiency of electron transport to the terminal acceptor of PSI, and this value was lower in T1 and T2 than in the CK (Fig 4G).

The performance index of PSII based on absorption (PI_abs_) sensitively reflects changes in the photosynthetic apparatus [27, 28]. As shown in Fig 4H, the PI_abs_ of T1 reached the highest on the 10^th^ day, and that of T2 and CK peaked on the 20^th^ day after the leaves had fully unfolded. Compared with the highest value in each group, the PI_abs_ decreased by 23.2% and 46.4% in T1, by 12.3% and 47.9% in T2, and by only 4.9% and 33.1% in CK, on the 40^th^ and 60^th^ day, respectively. From the first day to 20^th^ day, the PI_abs_ of T1 was higher than that of the CK, and a significant difference occurred on the 10^th^ day. However, the PI_abs_ of T1 was lower than that of the CK from the 40^th^ day to the 60^th^ day, with no significant difference. The PI_abs_ of T2 was higher than that of the CK from the first day to the 40^th^ day after the leaves had fully unfolded, and a significant difference occurred on the 10^th^ day and the 20^th^ day. On the 60^th^ day, the PI_abs_ of the CK was 10.7% and 6.8% higher than that of the T1 and T2, respectively. Based on analysis of the period from full leaf opening to yellowing, the PI_abs_ of T1 and T2 increased by 7.5% and 15.7%, respectively, in comparison with that of the CK.

### Effects of SL on chlorophyll content

**Fig 5:**
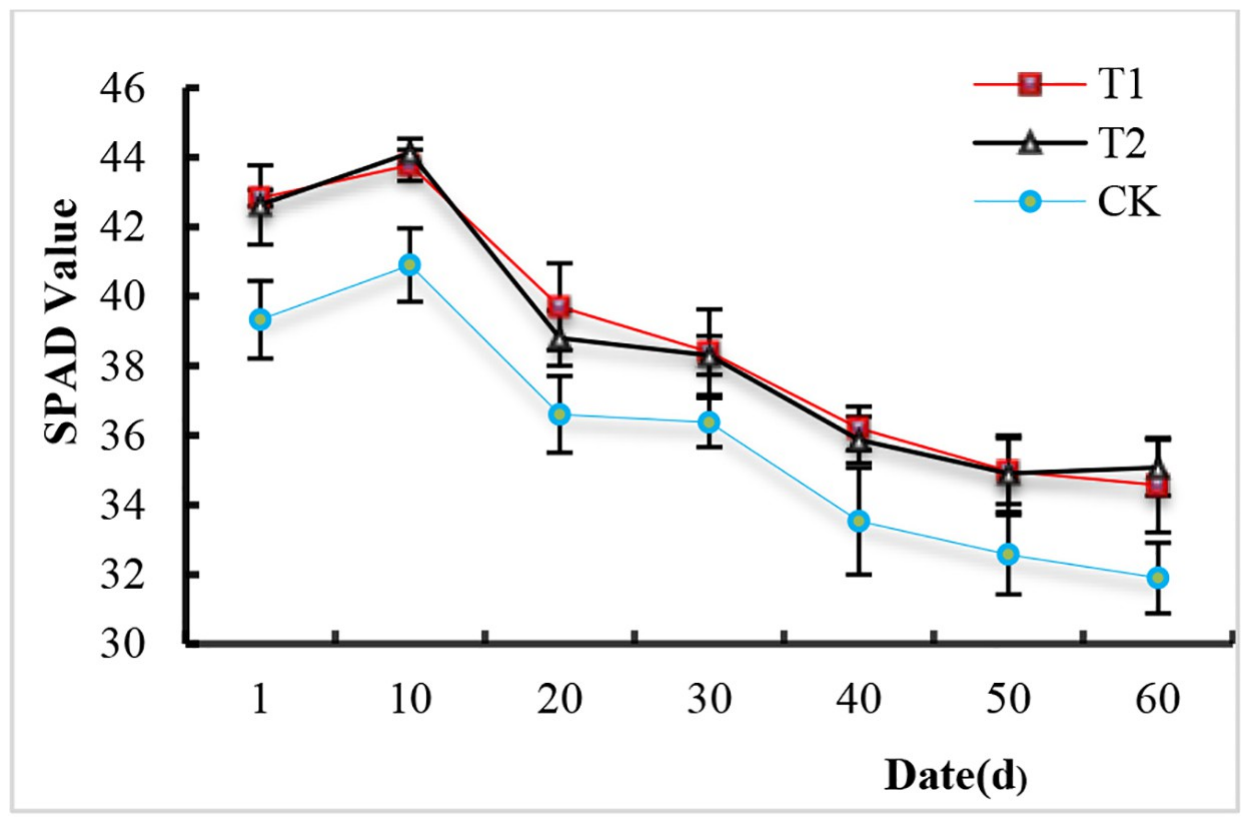
Effects of supplemental light on the chlorophyll content of tomato leaves. The values are the means (n = 3) ±SEs.

Fig 5 shows the change in chlorophyll content (SPAD value) from when the leaves were fully unfolded to when they turned yellow. The SPAD values were greatest on the 10^th^ day after unfolding and gradually decreased afterward. The chlorophyll content of all three treatments decreased to the lowest values on the 60^th^ day when the leaves began to turn yellow. In comparison with that of the CK treatment, the chlorophyll content of T1 and T2 significantly increased, and there was no significant difference between T1 and T2.

### Effects of SL on plant morphology

Table 3 shows that the SL altered the plant morphology, including the plant height, inter-node length, stem thickness and node on which the first flower occurred. The plants in T1 and T2 were significantly taller than those in CK. The ranking of the first node with a flower was T1>T2>CK, where the node in T1 was significantly higher than that in CK. Compared with that of plants in the CK, the stem thickness of the plants in T2 increased, while the stem thickness of the plants in T1 decreased. Notably, the difference in stem thickness between T2 and T1 was significant. Even in the absence of a significant difference, it was observed that SL could increase both the number of leaves and the inter-node length.

**Table 3:** Effects of SL on tomato plant morphology tomato.

	T1	T2	CK
Plant height (cm)	161.33±8.35^a^	156.9±6.42^a^	151.65±6.78^b^
Leaf number	16.95±1^a^	16.55±0.83^a^	16.40±0.94^a^
Inter-node length (cm)	10.13±0.48^a^	10.11±0.56^a^	9.88±0.67^a^
Node at which the first flower occurs (leaf number)	6.85±0.59^a^	6.5±0.51^ab^	6.35±0.67^b^
Stem diameter (mm)	12.46±0.98^b^	13.41±1.16^a^	13.11±1.07^ab^

1. Data were measured approximately 90 days after transplantation. 2. The values are the means (n = 20) ±SEs. Values followed by the same letters within a row are not significantly different at *p<0*.*05*.

### Impacts of SL on yield and quality

Among the treatment groups, T2 had the highest yield, which was 9.53% greater than that of the CK and significantly differed. Moreover, the yield of T1 was 4.14% higher than that of the CK, although they were not statistically different. The content of soluble sugar and titratable acidity determine the taste of tomato fruits. T2 fruits had the best quality, as their soluble sugar content and titratable acidity were significantly higher than those of the CK fruits, and there was no significant difference in these parameters between the SL treatments. The soluble sugar content and the titratable acidity content of the T1 fruits was comparable to that of CK fruits and did not significantly differ. In conclusion, T2 plants had the highest yield, and their fruits had the best quality, followed by those of T1 (Table 4).

**Table 4:** Effects of supplemental light on tomato yield and fruit quality.

		T1	T2	CK
Yield	Total yield (t ha^-1^)	83.01±3.1^ab^	87.31±5.56^a^	79.71±1.48^b^
	Percent increase (%)	4.14	9.53	
Quality	Soluble sugar content (%)	2.41±0.19^ab^	2.64±0.15^a^	2.1±0.28^b^
	Titratable acidity (%)	0.248±0.034^ab^	0.251±0.008^a^	0.21±0.004^b^

The values are the means (n = 3) ±SEs. Values followed by the same letters within a row are not significantly different at *p<0*.*05*.

### Effects of SL on color-changing and ripening of fruit

**Fig 6:**
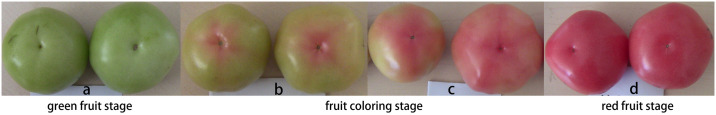
Color change and maturity of tomato fruits.

Color is the most important visible sign for judging tomato maturity. The L*, a*, and b* values measured by a colorimeter are used to quantify color changes. Lopez Camelo and Gómez [29] reported that a*/b* is a good criterion for tomato maturity grading. Fig 6 shows how changes in a*/b* values can be calibrated with color change and ripening of Kaide 6810 tomato fruits. During the green ripening stage, the a*/b* values were ≤-0.12 (Fig 6a) and ranged from -0.11 to 0 (Fig 6b) when the color started to change. Through the color-changing period, the a*/b* values were between 0 and 0.80 (Fig 6c) and ultimately surpassed 0.81 when the color change ended and the fruit ripened (Fig 6d).

Table 5 shows that at 100 days after transplanting, the first fruits on the first truss in T1 presented a a*/b* values ≥ 0.1, which indicates that the fruits were in the color-changing period. Moreover, the fruits in T2 were at the beginning of the color-changing period (-0.11< a*/b* <0), while those in the CK were at the green maturity stage (a*/b*≤-0.12). Ten days later, the a*/b* values of the fruits in T1 reached the calibrated harvestable value, and those in T2 and the CK were still associated with the color-changing stage. The percentages of ripe red fruits at both 100 days and 110 days after transplanting were in the order of T1>T2>CK, and there was a significant difference among the treatments. The red ripe fruit rate in T1 at 100 days was 48.3%, which was slightly higher than that in T2 at 110 days, and the red ripe fruit rate in T2 at 100 days was more than twice that in the CK at 110 days. These observations indicate that the first fruits on the first truss in T1 matured 10 days earlier than did those in T2, and those in T2 matured 10 days earlier than those in the CK did. Therefore, SL, especially that provided by HPS lamps, accelerates the color change and maturity of tomato fruits.

**Table 5:** Effects of supplemental light on color change and maturity of tomato fruits.

Date		T1	T2	Ck
100 days after transplantation	a*/b*	0.144±0.07^a^	-0.047±0.09^b^	-0.155±0.08^b^
	Percentage of red fruit (%)	48.3±0.01^a^	28.6±0.05^b^	3.1±0.04^c^
	Percentage of coloring fruit (%)	23.2±0.04^a^	17.3±0.10^a^	28.1±0.09^a^
	Percentage of green fruit (%)	28.5±0.04^b^	54.1±0.14^a^	68.8±0.12^a^
110 days after transplantation	a*/b*	0.846±0.11^a^	0.497±0.20^b^	0.209±0.08^c^
	Percentage of red fruit (%)	64.27±9.54^a^	44.20±13.63^b^	12.60±4.93^c^
	Percentage of coloring fruit (%)	32.40±6.58^b^	37.95±17.60^b^	71.77±11.42^a^
	Percentage of green fruit (%)	3.32±3.11^b^	17.85±9.76^a^	15.29±5.95^ab^

The values are the means (n = 3) ±SEs. Values followed by the same letters within a row are not significantly different at *p<0*.*05*.

## Discussion

Chlorophyll plays a key role in light absorption, and photosynthetic capacity increases with increasing chlorophyll content [30–32]. Jiang et al. [30] reported that the chlorophyll content of new leaves gradually increased with the development of leaves, and the net photosynthetic rate (Pn) increased at the same time. Sitko et al. [26] found that the maximum photosynthetic rate of young grape leaves occurred one week after their chlorophyll content peaked. In the present study, the maximum chlorophyll content occurred on the 10^th^ day after the leaves fully unfolded, and the ABS/CS ratio also concurrently peaked. However, the chlorophyll content gradually decreased beginning on the 20^th^ day, while the ABS/CS ratio was relatively stable. Tewolde et al. [20] reported that LED intralighting significantly increased the chlorophyll content in both middle and lower tomato canopy leaves. In our study, we showed that both HPS and LED supplemental lighting significantly increased the chlorophyll content in tomato leaves from when they fully unfolded to when they turned yellow (Fig 5). In terms of light energy absorption, we found that the ABS/CS ratio of the SL-treated group was obviously higher than that of the CK only from the 10^th^ day to the 40^th^ day after the leaves fully unfolded, although the differences were not significant on the first day or the 60^th^ day (Fig 4). Therefore, a higher chlorophyll content does not necessarily imply a higher ABS/CS value.

The ABS/CS ratio is determined by the ABS/RC and RC/CS ratios. Light growing conditions have a large effect on the antenna size of PSII (ABS/RC) [33–35]. Wientjes et al. [35] reported that the antenna size of PSII was smaller and the overall trapping time of PSII shorter when *Arabidopsis thaliana* was grown in high light (800 μmol m^−2^ s^− 1^). The opposite is observed in low light (20 μmol m^−2^ s^−1^). As a result, the value of quantum efficiency of charge separation decreased from 91% in high light to 84% in low light, which showed that the increased light absorption cross section of the large PSII antenna in low light plants came at the cost of decreased charge-separation efficiency. In our study, We also observed that the ABS/RC and RC/CS ratios did not show statistically significant differences between the SL-treated groups and the CK group. However, by comparing the two SL-treated groups, we found a slightly higher ABS/RC ratio in the HPS lamp-treated groups and a slightly higher RC/CS ratio in the LED-treated groups. This might indicate that treatment of plants with HPS-SL increased the light absorption cross section due to a larger antenna size of PSII (higher ABS/RC ratio), but compared with plants treated with LED-SL which perhaps have a smaller antenna size and a faster overall PSII trapping time due to higher RC/CS ratio, the quantum yield of electron transport (φ_Eo_) in plants treated with HPS light was downregulated by approximately 1.2%. The RE_0_/RC ratio of the young leaves (1–20 days after leaf unfolding) in the SL-treated groups did not show a significant difference from that in the CK group but was significantly lower than that in the CK on the 40^th^ day (Fig 4). At the same time, the ΔV_K_ and ΔV_J_ values of the SL-treated groups were significantly higher than that of the CK from the 40^th^ to 60^th^ days (Fig 3), which implied that the decrease in OEC activity and the increase in inhibition of Q_A_ to Q_B_ electron transport occurred simultaneously with the lower RE_0_/RC ratio. These results could be caused by aging and response to stress [26, 36].

Gómez and Mitchell [4] applied three different SL strategies for growing tomato fruits in winter, namely, overhead canopy HPS lamps vs. intracanopy LEDs vs. a hybrid of overhead HPS lamp and intracanopy LEDs, with the goal of achieving a total DLI of 25 mol m^-2^ day^-1^. The F_v_/F_m_ ratio did not differ substantially between the SL-treated groups and the CK group. However, the Pn of the leaves, especially in the intracanopy LED- and hybrid-treated groups, was significantly higher than that in the CK, and the yield increased by 33%. In our study, the F_v_/F_m_ (=φ_Po_) value between the SL-treated groups and the CK group was also not significantly different from when the leaves were fully unfolded to when they turned yellow. The φ_Eo_ and PI_abs_ values of the LED-treated group were the highest, followed by those in the HPS lamp-treated group and the CK group. Therefore, based on the performance of the photosynthetic apparatus, we conclude that the SL treatment improved the ABS/CS, φ_Eo_ and PI_abs_ values of the leaves, which increased the photosynthetic capacity accordingly, and enhanced the yield and fruit quality. The increase in fruit fresh weight and amount of supplemental lighting showed a positive linear relationship [19]. In the present study, compared with the CK group, the LED-treated group presented a 9.53% greater yield, which was significantly different, while the yield of the HPS lamp-treated group was only 4.14% higher and did not significantly differ (Table 4). Tewolde et al. [20] reported that RB-LED lighting treatment for 10 h in the winter increased the soluble solid content and titratable acidity of tomato fruits by 20% and 25%, respectively. Similarly, Lu et al. [19] applied intracanopy lighting at different developmental stages of single-truss tomato plants, and the sugar content increased to 12% (Brix%). Lu and Mitchell [8] used a LED-SL strategy, which successfully increased the sugar content of tomato fruits by 11–12%. In our study, the soluble sugar content of the HPS- and LED-treated groups increased by 14.8% and 25.7%, respectively, and the titratable acidity increased by 18.1% and 19.5%, respectively (Table 4).

Appenroth et al. [27] and Van Heerden et al. [28] pointed out that, instead of the F_v_/F_m_ ratio, the PI_abs_ can more precisely reflect the state of the photosynthetic apparatus. This study showed that the plants in the groups treated with SL presented their maximal PI_abs_ value earlier than plants in the CK group, which implies that SL accelerated the formation of the PSII apparatus. Dueck et al. [37] found that, in comparison with those under LED supplemental lighting, the leaves of tomato plants grown under HPS lamp-provided supplemental lighting were thinner and aged more rapidly in winter. Our results showed that the plants were taller and the stem thickness was reduced following the HPS treatment compared with the LED treatment. This may be related to the greater amount of heat generated by the HPS lamps, in addition to the slightly higher temperature and faster leaf development. At the same time, our results show that the PI_abs_ of leaves grown under HPS lamp dropped rapidly from the 20^th^ day on after unfolding, but that this rapid drop in the LED-treated group occurred from the 40^th^ to 60^th^ day, which was more obvious than that in the CK in leaves of the same age (Fig 4). In association with the ΔV_K_ and ΔV_J_ values from the OJIP curve, we assume that the leaves under the HPS lamps aged the most rapidly, followed by those under the LED lighting and those exposed to the CK treatment.

In their study, Lee et al. [15] applied 80-W RB-LED-based SL, and the fruits of tomato were harvested 17 days earlier than those of the CK; moreover, the authors reported harvesting 7 days earlier when a 200-W HPS lamp-based SL was used. Gómez et al. [38] studied the effects of overhead HPS lamps and intra-canopy LEDs on tomato fruit ripening; an early harvest—by 24 days (HPS) and 22 d (LED)—was observed when 9 mol m^-2^ day^-1^ DLI supplemental lighting was applied. In our study, the a*/b* ratio and the red ripe fruit rate were used to evaluate the effects of SL on fruit color changes and ripening. Compared with those in the CK group, the fruits in the HPS lamp- and LED-treated groups ripened 20 days and 10 days earlier, respectively (Table 5). We believe that early maturity is closely related to both early leaf development and early aging.

## Conclusion

Compared with the SL strategies used in other studies, our strategy involves reducing the number of light sources (total power of 30.6 W m^-2^) and shortening the photoperiod of SL (3 h day^-1^), therefore, the energy consumption required for the SL is relatively low (<0.1 kWh m^-2^ day^-1^). However, this treatment promotes the performance of the leaf photosynthetic apparatus, promotes plant growth and increases yield. Although the yield and fruit quality obtained via the LED SL strategy is better than that resulting from the HPS-SL strategy, it is desirable for the fruit to change color and for maturity to occur 10 days earlier, as observed in plants grown under HPS-SL compared with plants grown under LED SL.

## Supporting information

S1 Dataset (XLSX)
